# Supplementary figures and images for: Impact of crop residue management on crop production and soil chemistry after seven years of crop rotation in temperate climate, loamy soils
Source: PeerJ. 2018 May 23;6:e4836. doi: 10.7717/peerj.4836 (PMC5970559; doi:10.7717/peerj.4836)

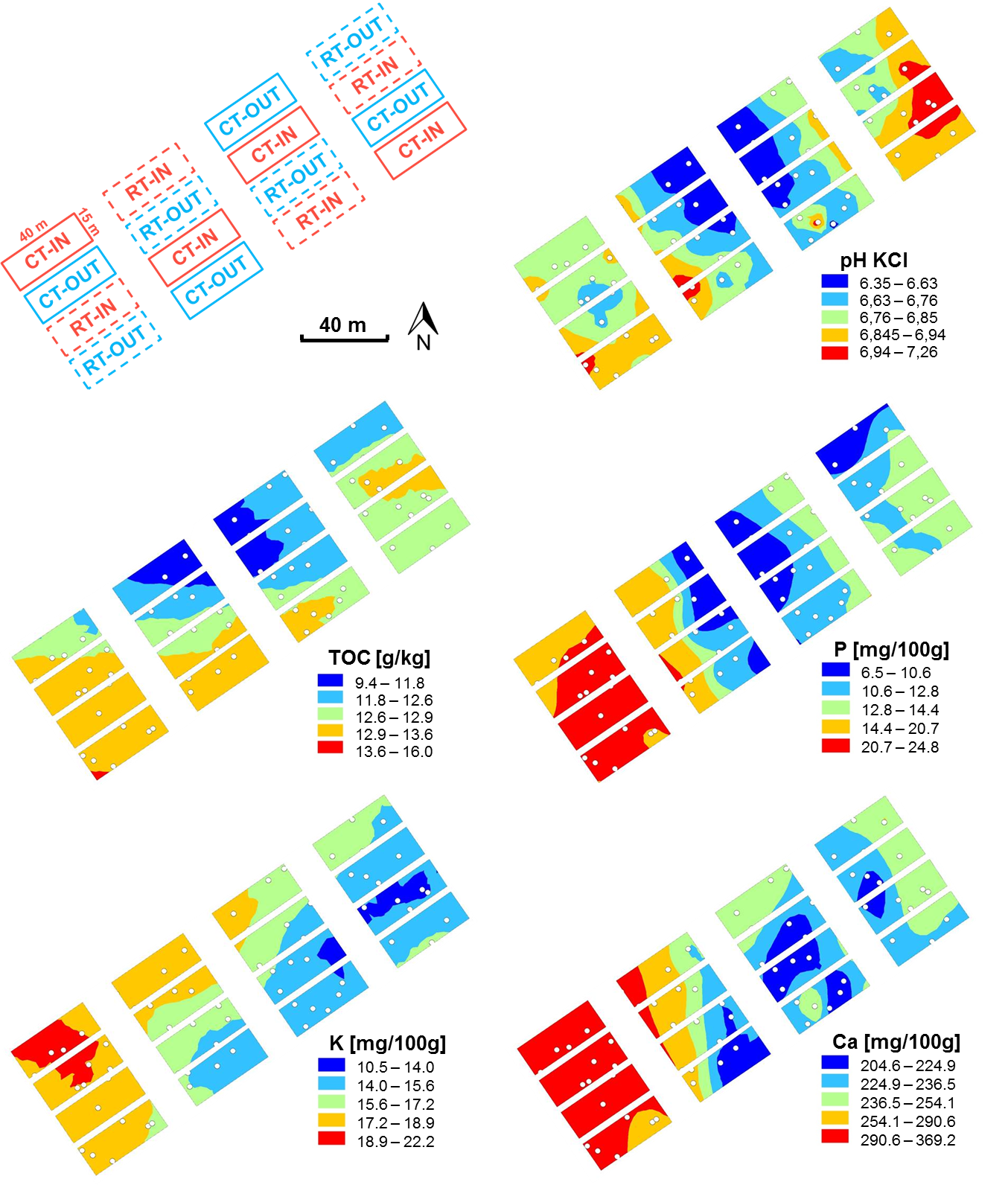

Supplement: Figure S1 — Data were collected in 2011. Maps were obtained by interpolating using the kriging methods in ArcGIS. [file peerj-06-4836-s001.png]

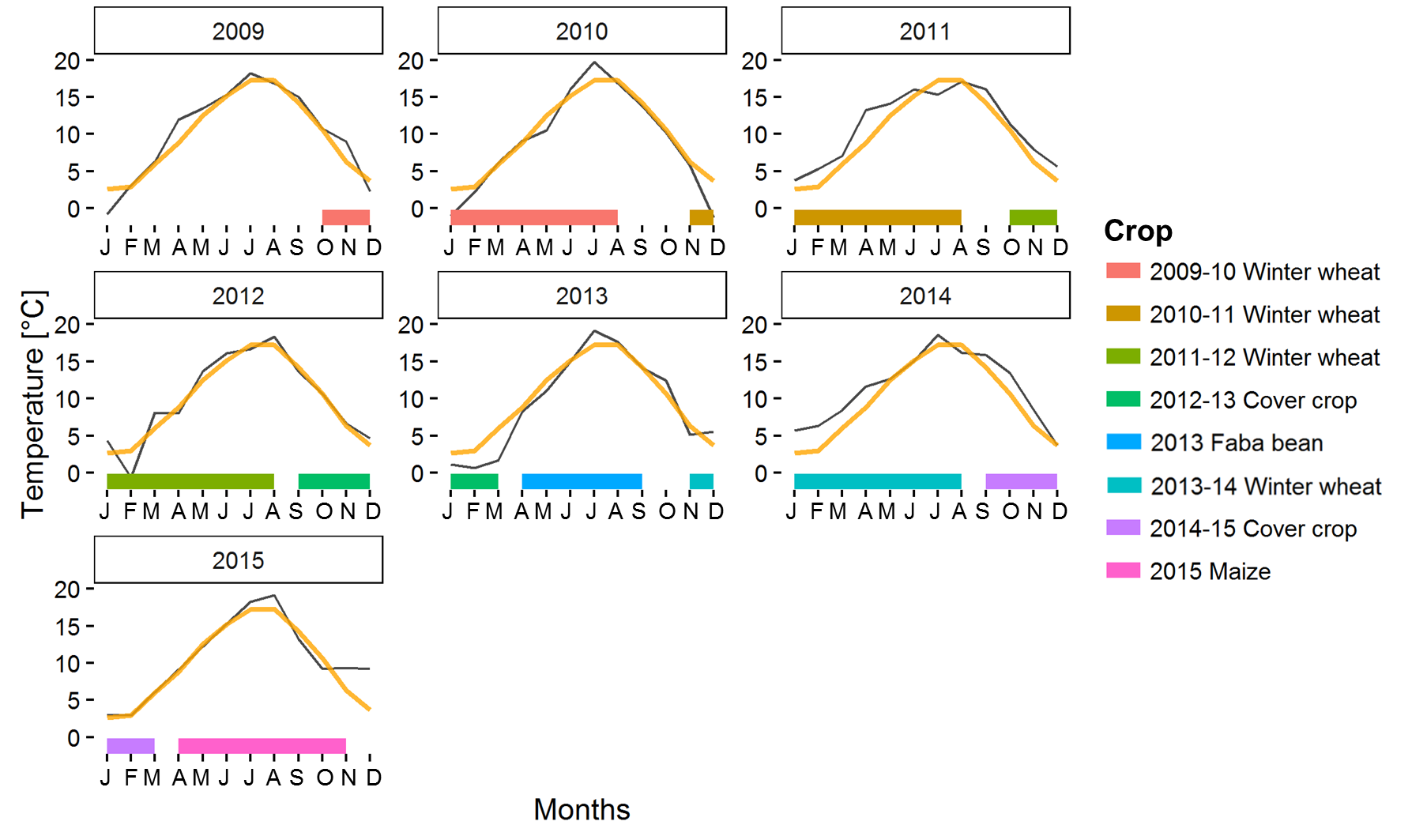

Supplement: Figure S2 — Orange line is the 30 year mean, grey line is the observations for each year. [file peerj-06-4836-s002.png]

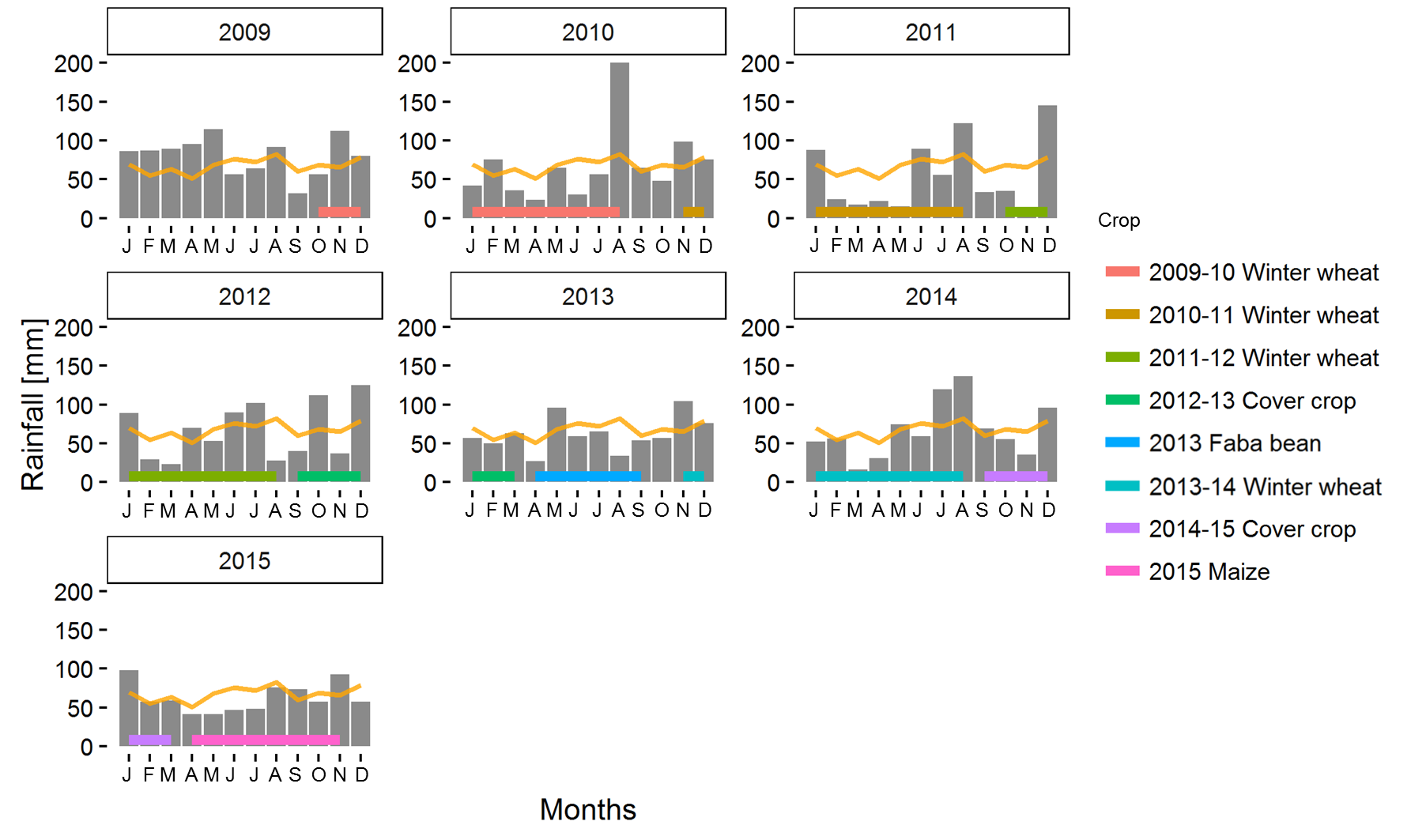

Supplement: Figure S3 — Orange line is the 30 year mean, grey bars show the observation for the year. [file peerj-06-4836-s003.png]

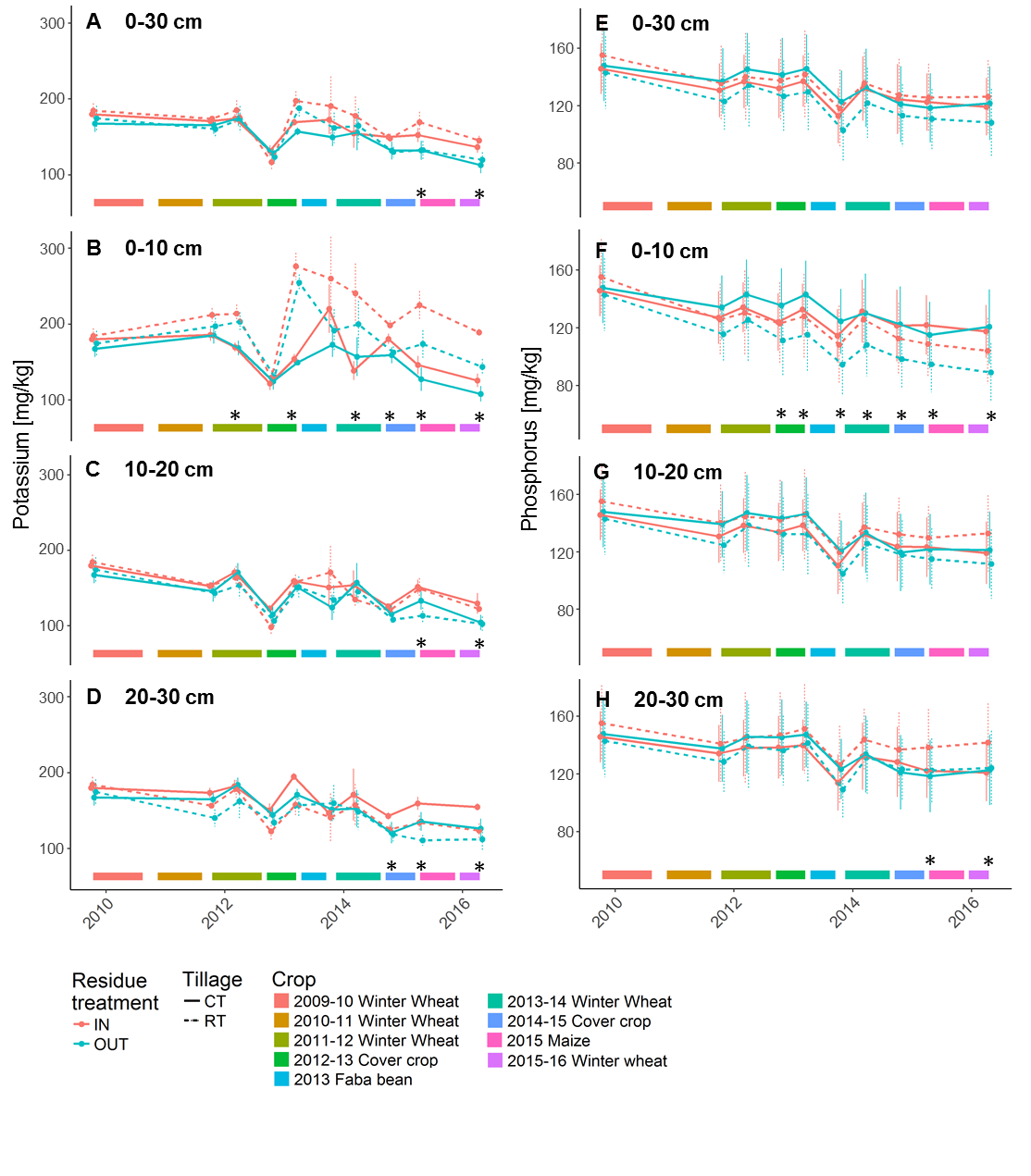

Supplement: Figure S4 — Bottom ribbons represent the period covered by crops. Error bars depict the standard error between plots. Stars represent significant differences (ANOVA, p-value <0.05) between crop residue management strategies per sampling date. [file peerj-06-4836-s004.png]

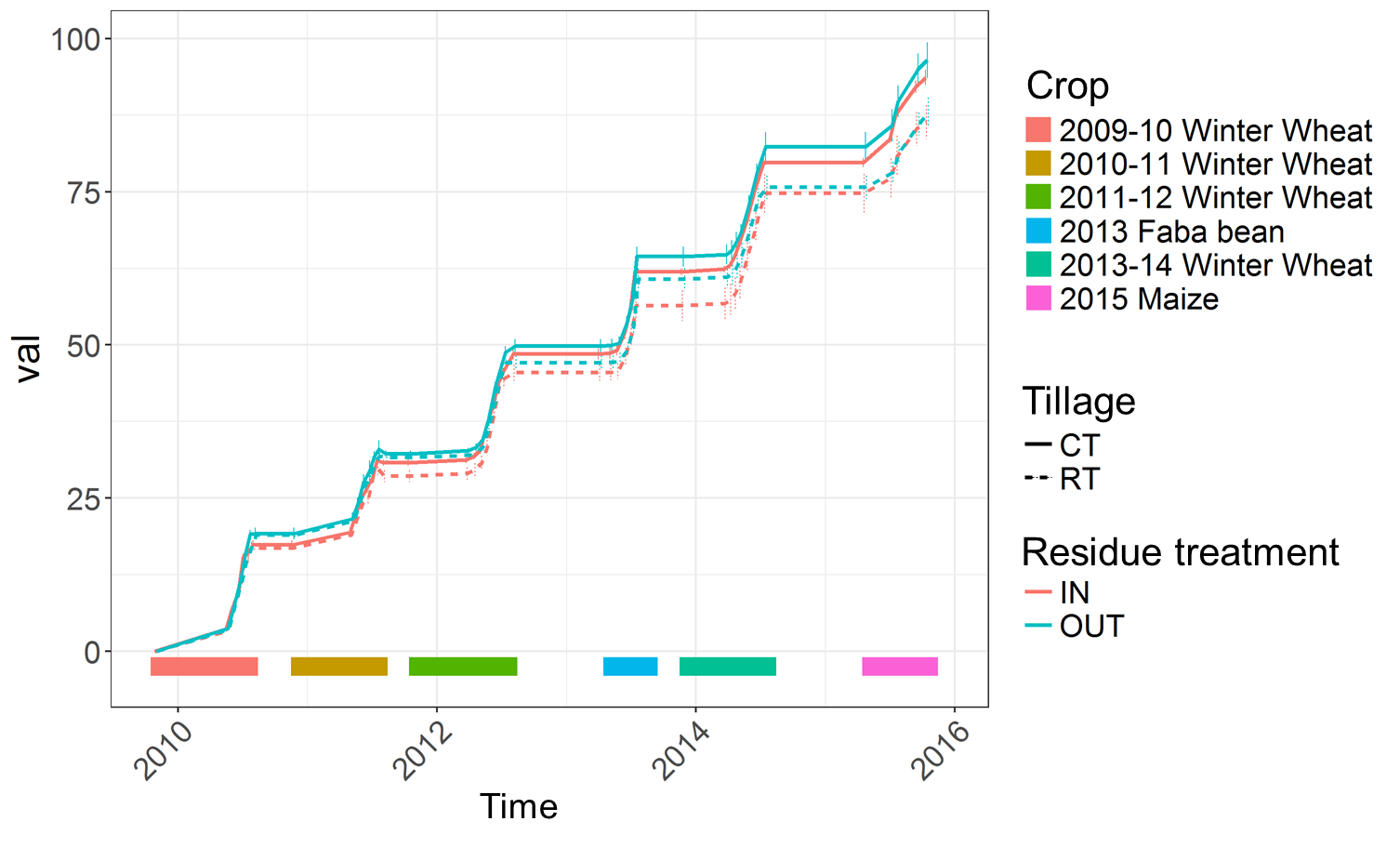

Supplement: Figure S5 — Error bars depict the standard error between plots. [file peerj-06-4836-s005.png]
