# Supplementary material for: Impact of crop residue management on crop production and soil chemistry after seven years of crop rotation in temperate climate, loamy soils
Source: PeerJ. 2018 May 23;6:e4836. doi: 10.7717/peerj.4836 (PMC5970559; doi:10.7717/peerj.4836)
Supplement: Table S1 [file peerj-06-4836-s006.docx]

| Date | operation | depth (cm) | Additional information | CT-IN | CT-OUT | RT-IN | RT-OUT |
| --- | --- | --- | --- | --- | --- | --- | --- |
| 3/09/2008 | weeding | 0 | with Roundup (6.43 L/ha) | x | x | x | x |
| 8/09/2008 | stubble breaking | –10 | With stubble cultivator (Lemken Smaragd 9/300) | x | x | x | x |
| 11/09/2008 | ploughing | –25 | with mouldboard plough | x | x |  |  |
| 11/09/2008 | sowing | –7 | rapeseed | x | x | x | x |
| 13/10/2008 | weeding |  |  | x | x | x | x |
| 13/03/2009 | nitrogen fertilisation |  | liquid nitrogen (39%), 86 kg/ha of nitrogen | x | x | x | x |
| 2/04/2009 | nitrogen fertilisation |  | liquid nitrogen (39%), 80 kg/ha of nitrogen | x | x | x | x |
| 3/04/2009 | fungicide |  | application of Caramba 60 SL (1.2 L/ha) | x | x | x | x |
| 4/04/2009 | insecticide |  | application of Biscaya (0.3 L/ha) | x | x | x | x |
| 24/04/2009 | fungicide |  | application of Cantus (0.5 kg/ha) | x | x | x | x |
| 31/07/2009 | harvest |  | harvest of rapeseed crop | x | x | x | x |
| 31/07/2009 | residue exportation |  | with a tray at the end of the combine |  | x |  | x |
| 18/08/2009 | stubble breaking | –10 | With stubble cultivator (Lemken Smaragd 9/300) | x | x | x | x |
| 21/09/2009 | stubble breaking | –10 | With stubble cultivator (Lemken Smaragd 9/300) | x | x | x | x |
| 30/10/2009 | ploughing | –25 | with mouldboard plough | x | x |  |  |
| 30/10/2009 | sowing |  | with winter wheat (Lear, 135 kg/ha), the tractor was equipped with a dual cultivator (Jadin) in front and rotary harrow and wedge ring roller combined with seed drill (Amazone) | x | x | x | x |
| 8/04/2010 | nitrogen fertilisation |  | liquid nitrogen (39%), 80 kg/ha of nitrogen | x | x | x | x |
| 14/04/2010 | weeding |  | application of Atlantis WG (0.30 kg/ha), Milan (1.25 L/ha), Primus (0.05 L/ha) and Vegetop (1 L/ha) | x | x | x | x |
| 29/04/2010 | growth regulator |  | application of Cycofix (1 L/ha) | x | x | x | x |
| 28/05/2010 | nitrogen fertilisation |  | liquid nitrogen (39%), 100 kg/ha of nitrogen | x | x | x | x |
| 24/06/2010 | fungicide |  | application of Fandango Pro (2 L/ha) | x | x | x | x |
| 1/08/2010 | harvest |  | harvest of winter wheat with experimental combine | x | x | x | x |
| 1/08/2010 | residue exportation |  | straw bale exported out of the field |  | x |  | x |
| 8/09/2010 | stubble breaking | –10 | With stubble cultivator (Lemken Smaragd 9/300) | x | x | x | x |
| 8/09/2010 | stubble breaking | –10 | with stubble cultivator (Jadin) | x | x | x | x |
| 22/11/2010 | ploughing | –25 | with mouldboard plough | x | x |  |  |
| 22/11/2010 | sowing | –7 | with winter wheat (Sahara, 175 kg/ha), the tractor was equipped with a dual cultivator (Jadin) in front and rotary harrow and wedge ring roller combined with seed drill (Amazone) | x | x | x | x |
| 15/03/2011 | nitrogen fertilisation |  | liquid nitrogen (39%), 70 kg/ha of nitrogen | x | x | x | x |
| 13/04/2011 | weeding |  | application of Othello (1.2 L/ha) and Legacy (0.2 L/ha) | x | x | x | x |
| 15/04/2011 | nitrogen fertilisation |  | liquid nitrogen (39%), 50 kg/ha of nitrogen | x | x | x | x |
| 22/04/2011 | growth regulator |  | application of Cycocel (1 L/ha) | x | x | x | x |
| 20/05/2011 | insecticide |  | application of Karate (0.05 L/ha) | x | x | x | x |
| 25/05/2011 | nitrogen fertilisation |  | liquid nitrogen (39%), 60 kg/ha of nitrogen | x | x | x | x |
| 20/08/2011 | harvest |  | harvest of winter wheat with experimental combine | x | x | x | x |
| 20/08/2011 | residue exportation |  | straw bale exported out of the field |  | x |  | x |
| 9/09/2011 | stubble breaking | –10 | With stubble cultivator (Lemken Smaragd 9/300) | x | x | x | x |
| 29/09/2011 | stubble breaking | –10 | With stubble cultivator (Lemken Smaragd 9/300) | x | x | x | x |
| 17/10/2011 | ploughing | –25 | with mouldboard plough | x | x |  |  |
| 17/10/2011 | sowing | –7 | with winter wheat (Sahara, 135 kg/ha), the tractor was equipped with a dual cultivator (Jadin) in front and rotary harrow and wedge ring roller combined with seed drill (Amazone) | x | x | x | x |
| 23/03/2012 | nitrogen fertilisation |  | liquid nitrogen (39%), 50 kg/ha of nitrogen | x | x | x | x |
| 28/03/2012 | weeding |  | application of Othello (1.2 L/ha) and Legacy (0.4 L/ha) | x | x | x | x |
| 3/05/2012 | growth regulator |  | application of Cycofix (1 L/ha) | x | x | x | x |
| 14/05/2012 | nitrogen fertilisation |  | liquid nitrogen (39%), 60 kg/ha of nitrogen | x | x | x | x |
| 15/05/2012 | fungicide |  | application of Palazzo (2 L/ha) | x | x | x | x |
| 5/06/2012 | nitrogen fertilisation |  | solid nitrogen, 75 kg/ha of nitrogen | x | x | x | x |
| 14/06/2012 | fungicide |  | application of Aviator (1.25 L/ha) | x | x | x | x |
| 13/08/2012 | harvest |  | harvest of winter wheat with experimental combine | x | x | x | x |
| 13/08/2012 | residue exportation |  | straw bale exported out of the field |  | x |  | x |
| 29/08/2012 | stubble breaking | –10 | with stubble cultivator (Jadin) | x | x | x | x |
| 6/09/2012 | cover crop sowing | –7 | with mustard CHACHA / ABA anti-nematode (10 kg/ha) | x | x | x | x |
| 13/12/2012 | ploughing | –25 | with mouldboard plough | x | x |  |  |
| 18/03/2013 | weeding |  | Glyphosate (2.59 L/ha) | x | x | x | x |
| 5/04/2013 | sowing | –7 | with faba bean, variety Espresso (300 kg/ha) | x | x | x | x |
| 8/04/2013 | weeding |  | pre-emergence weeding with application of Lingo (1.4 L/ha) and Stomp (1.8 L/ha) | x | x | x | x |
| 10/06/2013 | weeding |  | manually only on thistle with application of a herbicide | x | x | x | x |
| 8/07/2013 | insecticide |  | because of the presence of some black aphids and weevils (not as many), application of Karate Zeon (0.05 L/ha) | x | x | x | x |
| 28/08/2013 | weeding |  | application of Diquanet SL | x | x | x | x |
| 4/09/2013 | harvest |  | harvest of faba bean with experimental combine | x | x | x | x |
| 4/09/2013 | residue exportation |  | with a tray at the end of the combine |  | x |  | x |
| 25/11/2013 | ploughing | –25 | with mouldboard plough | x | x |  |  |
| 25/11/2013 | sowing | –7 | with winter wheat (Edgard, 300 kernels/m²), the tractor was equipped with a dual cultivator (Jadin) in front and rotary harrow and wedge ring roller combined with seed drill (Amazone) | x | x | x | x |
| 11/03/2014 | nitrogen fertilisation |  | liquid nitrogen (39%) 95.07 L/ha which is 37 kg/ha of nitrogen | x | x | x | x |
| 1/04/2014 | weeding |  | application of Atlantis (0.3 kg/ha), Hussar Ultra (0.1 L/ha) and Actirob B (1 L/ha) | x | x | x | x |
| 15/04/2014 | nitrogen fertilisation |  | liquid nitrogen (39%) 157.73 L/ha which is 62 kg/ha of nitrogen | x | x | x | x |
| 15/04/2014 | growth regulator |  | application of Cycofix 750G (1.02 L/ha) | x | x | x | x |
| 25/04/2014 | weeding |  | application of Axial (1.47 L/ha) | x | x | x | x |
| 27/04/2014 | fungicide |  | application of Osiris (2.03 L/ha) | x | x | x | x |
| 12/05/2014 | nitrogen fertilisation |  | liquid nitrogen (39%) 181.54 L/ha which is 72 kg/ha of nitrogen | x | x | x | x |
| 16/05/2014 | weeding |  | application of Allie (30.55 g/ha) | x | x | x | x |
| 6/06/2014 | fungicide |  | application of Aviator (1.27 L/ha) | x | x | x | x |
| 4/09/2014 | harvest |  | harvest of winter wheat | x | x | x | x |
| 4/09/2014 | residue exportation |  | straw bale exported out of the field |  | x |  | x |
| 12/09/2014 | stubble breaking | –10 | 10 cm depth with stubble cultivator (Jadin) | x | x | x | x |
| 16/09/2014 | cover crop sowing |  | with oats (40 kg/ha) with Jadin tool in front of tractor and sowing machine combined with harrow at the back and peas (65 kg/ha) in a second passage with only the sowing machine | x | x | x | x |
| 6/01/2015 | ploughing | –25 | with mouldboard plough | x | x |  |  |
| 17/03/2015 | weeding |  | application of glyphosate (4.16 L/ha) | x | x | x | x |
| 20/04/2015 | nitrogen fertilisation |  | liquid nitrogen (39%) 313.86 L/ha which is 122 kg/ha of nitrogen | x | x | x | x |
| 22/04/2015 | Shallow tillage | -10 | with dual cultivator (Jadin) in front of tractor and harrow at the back combined with crosskill roller (Amazone) | x | x | x | x |
| 22/04/2015 | sowing | -5 | with corn maize (LG 30.215) | x | x | x | x |
| 28/05/2015 | weeding |  | application of Andes (1.6 L/ha), Callisto (0.71 L/ha) and Samson extra 6 (0.42 L/ha) | x | x | x | x |
| 13/11/2015 | harvest |  | with experimental combine | x | x | x | x |
| 16/11/2015 | residue exportation |  | Manually |  | x |  | x |
