# Supplementary material for: Impact of crop residue management on crop production and soil chemistry after seven years of crop rotation in temperate climate, loamy soils
Source: PeerJ. 2018 May 23;6:e4836. doi: 10.7717/peerj.4836 (PMC5970559; doi:10.7717/peerj.4836)
Supplement: Table S2 — Significance code: ‘***’ p-value < 0.001; ‘**’ p-value < 0.01; ‘*’ p-value < 0.05. (Df: degree of freedom, Mean Sq: mean square). [file peerj-06-4836-s007.docx]

| **Df Mean Sq Fvalue Pvalue** |
| --- |
| Rapeseed 2008-09 Tillage 1 0.56 1.937 0.213  Residue 1 188.50 650.744 2.39e-07 ***  Tillage*Residue 1 0.21 0.719 0.429  WW 2009-10 Tillage 1 2.356 11.957 0.013502 *  Residue 1 10.989 55.766 0.000298 ***  Tillage*Residue 1 0.255 1.294 0.298663  WW 2010-11 Tillage 1 2.681 1.104 0.3338  Residue 1 25.680 10.573 0.0174 *  Tillage*Residue 1 0.761 0.313 0.5958  WW 2011-12 Tillage 1 0.04 0.030 0.867790  Residue 1 59.46 45.377 0.000521 ***  Tillage*Residue 1 0.01 0.007 0.935810  Cover crop 2012-13 Tillage 1 3.210 118.872 3.53e-05 ***  Residue 1 0.069 2.572 0.1599  Tillage*Residue 1 0.001 0.055 0.8223  Faba2013 Tillage 1 1.57 3.530 0.109  Residue 1 43.73 98.317 6.08e-05 ***  Tillage*Residue 1 0.04 0.092 0.772  WW2013-14 Tillage 1 0.03 0.011 0.91863  Residue 1 91.06 29.548 0.00161 **  Tillage*Residue 1 0.54 0.176 0.68942  Cover crop 2014-15 Tillage 1 1.5544 22.869 0.00306 **  Residue 1 0.0047 0.069 0.80140  Tillage*Residue 1 0.0249 0.367 0.56705  Maize 2015 Tillage 1 2.35 2.574 0.160  Residue 1 140.29 153.640 1.68e-05 ***  Tillage*Residue 1 2.83 3.102 0.129 |
| **TOTAL Tillage 1 9 1.241 0.308**  **Residue 1 3321 437.114 7.8e-07** ***  **Tillage*Residue 1 3 0.416 0.543** |
