# Supplementary material for: Impact of crop residue management on crop production and soil chemistry after seven years of crop rotation in temperate climate, loamy soils
Source: PeerJ. 2018 May 23;6:e4836. doi: 10.7717/peerj.4836 (PMC5970559; doi:10.7717/peerj.4836)
Supplement: Table S3 — For each crop, treatments means with different letters are significantly different (ANOVA, p-value < 0.05). (WW: winter wheat, CT: conventional tillage, RT: reduced tillage, IN: incorporation of crop residue, OUT: exportation of crop residues). [file peerj-06-4836-s008.docx]

| Interaction between fixed factors No interaction between factors |
| --- |
| Nutrient Crop Crop residue management Residue fate Tillage type |
| CT-IN CT-OUT RT-IN RT-OUT IN OUT CT RT |
| N [kg/ha] WW2010-11 27.61 ± 6.27 14.69 ± 1.03 24.23 ± 2.58 13.01 ± 1.22 **25.92a ± 3.2 13.85b ± 0.8** 21.15a ± 3.82 18.62a ± 2.5  WW2011-12 43.48 ± 7.67 19.6 ± 2.08 45.07 ± 3.65 23.44 ± 1.44 **44.27a ± 3.95 21.52b ± 1.38** 31.54a ± 5.82 34.26a ± 4.47  Faba 2013 74.12 ± 6.12 46.58 ± 4.36 53.64 ± 4.39 40.48 ± 5.95 **63.88a ± 5.21 43.53b ± 3.6** 60.35a ± 6.26 47.06a ± 4.23  WW2013-14 45.76 ± 7.76 28.77 ± 2.64 51.57 ± 3.15 24.23 ± 2.9 **48.67a ± 4.03 26.5b ± 2.01** 37.26a ± 4.97 37.9a ± 5.53  Maize 2015 67.22 ± 8.46 20.95 ± 1.86 53.24 ± 2.75 23.69 ± 3.5 **60.23a ± 4.89 22.32b ± 1.91** 44.09a ± 9.62 38.47a ± 5.95  P [kg/ha] WW2010-11 3.99 ± 1.13 1.7 ± 0.1 2.7 ± 0.34 1.44 ± 0.18 **3.34a ± 0.6 1.57b ± 0.11** 2.84a ± 0.68 2.07a ± 0.3  WW2011-12 6.59 ± 1.17 3.37 ± 0.62 6.37 ± 0.74 3.59 ± 0.45 **6.48a ± 0.64 3.48b ± 0.36** 4.98a ± 0.86 4.98a ± 0.66  Faba 2013 10.37 ± 1.89 4.75 ± 0.91 6.82 ± 0.98 4.02 ± 0.65 **8.6a ± 1.19 4.38b ± 0.53** 7.56a ± 1.44 5.42a ± 0.76  WW2013-14 7.68 ± 1.34 4.44 ± 0.35 7.07 ± 0.11 3.61 ± 0.44 **7.37a** **± 0.63 4.02b ± 0.3** 6.06a ± 0.89 5.34a ± 0.69  Maize 2015 7.91 ± 0.46 2.72 ± 0.3 7.83 ± 0.43 3.23 ± 0.46 **7.87a ± 0.29 2.97b ± 0.27** 5.31a ± 1.01 5.53a ± 0.92  K [kg/ha] WW2010-11 15.26 ± 4.09 6.41 ± 0.78 12.32 ± 0.34 5.62 ± 0.95 **13.79a ± 1.98 6.01b ± 0.59** 10.83a ± 2.55 8.97a ± 1.35  WW2011-12 53.27 ± 5.01 16.42 ± 2.27 61.51 ± 6.3 18.22 ± 0.82 **57.39a ± 4.04 17.32b ± 1.17** 34.84a ± 7.42 39.86a ± 8.69  Faba 2013 41.78 ± 7.39 16.01 ± 1.07 42.72 ± 5.49 11.56 ± 1.61 **42.25a ± 4.27 13.78b ± 1.23** 28.89a ± 5.97 27.14a ± 6.46  WW2013-14 16.59 ± 2.77 7.88 ± 0.58 18.16 ± 1.01 7.08 ± 0.77 **17.38a ± 1.4 7.48b ± 0.47** 12.24a ± 2.11 12.62a ± 2.17  Maize 2015 104.29 ± 18.9 24.66 ± 1.23 86.9 ± 6.8 29.05 ± 2.5 **95.6a ± 9.86 26.85b ± 1.53** 64.47a ± 17.42 57.98a ±11.44 |
