# Supplementary material for: Impact of crop residue management on crop production and soil chemistry after seven years of crop rotation in temperate climate, loamy soils
Source: PeerJ. 2018 May 23;6:e4836. doi: 10.7717/peerj.4836 (PMC5970559; doi:10.7717/peerj.4836)
Supplement: Table S4 — Significance code: ‘***’ p-value < 0.001; ‘**’ p-value < 0.01; ‘*’ p-value < 0.05. (Df: degree of freedom, Mean Sq: mean square). [file peerj-06-4836-s009.docx]

| **Nutrient Crop Df Mean Sq Fvalue Pvalue** |
| --- |
| **N** WW2010-11 Tillage 1 25.6 0.405 0.5482  Residue 1 582.9 9.218 0.0229 *  Tillage*Residue 1 2.9 0.045 0.8383  WW2011-12 Tillage 1 29.5 0.671 0.443911  Residue 1 2070.4 47.191 0.000469 ***  Tillage*Residue 1 5.1 0.115 0.745566  Faba2013 Tillage 1 706.2 4.423 0.0801 .  Residue 1 1656.7 10.377 0.0181 *  Tillage*Residue 1 206.8 1.296 0.2984  WW2013-14 Tillage 1 1.6 0.013 0.91323  Residue 1 1965.7 15.579 0.00756 **  Tillage*Residue 1 106.9 0.847 0.39286  Maize2015 Tillage 1 126 1.190 0.317218  Residue 1 5750 54.110 0.000323 ***  Tillage*Residue 1 279 2.630 0.155974  **P** WW2010-11 Tillage 1 2.387 1.497 0.2670  Residue 1 12.572 7.885 0.0308 *  Tillage*Residue 1 1.078 0.676 0.4423  WW2011-12 Tillage 1 0.00 0.000 0.99739  Residue 1 36.12 27.232 0.00198 **  Tillage*Residue 1 0.20 0.148 0.71370  Faba2013 Tillage 1 18.40 2.151 0.193  Residue 1 71.03 8.301 0.028 *  Tillage*Residue 1 7.96 0.930 0.372  WW2013-14 Tillage 1 2.05 0.833 0.39648  Residue 1 44.90 18.220 0.00527 **  Tillage*Residue 1 0.05 0.020 0.89311  Maize2015 Tillage 1 0.19 0.234 0.646  Residue 1 95.98 121.189 3.34e-05 ***  Tillage*Residue 1 0.34 0.430 0.536  **K** WW2010-11 Tillage 1 13.93 0.992 0.358  Residue 1 242.06 17.241 0.006 **  Tillage*Residue 1 4.62 0.329 0.587  WW2011-12 Tillage 1 101 1.888 0.218  Residue 1 6422 120.302 3.41e-05 ***  Tillage*Residue 1 41 0.774 0.413  Faba2013 Tillage 1 12 0.077 0.79088  Residue 1 3241 20.329 0.00407 **  Tillage*Residue 1 29 0.182 0.68454  WW2013-14 Tillage 1 0.6 0.057 0.819735  Residue 1 391.5 37.415 0.000872 ***  Tillage*Residue 1 5.6 0.533 0.492862  Maize2015 Tillage 1 169 0.352 0.574539  Residue 1 18904 39.425 0.000759 ***  Tillage*Residue 1 475 0.990 0.358233 |
