# Supplementary material for: Impact of crop residue management on crop production and soil chemistry after seven years of crop rotation in temperate climate, loamy soils
Source: PeerJ. 2018 May 23;6:e4836. doi: 10.7717/peerj.4836 (PMC5970559; doi:10.7717/peerj.4836)
Supplement: Table S5 — For each crop, treatments means with different letters are significantly different (ANOVA, p-value < 0.05). (WW: winter wheat, CT: conventional tillage, RT: reduced tillage, IN: incorporation of crop residue, OUT: exportation of crop residues). [file peerj-06-4836-s010.docx]

| Interaction between fixed factors No interaction between factors |
| --- |
| Nutrient Crop Crop residue management Residue fate Tillage type |
| CT-IN CT-OUT RT-IN RT-OUT IN OUT CT RT |
| N [g/kg] WW 2010-11 4.7 ± 0.38 5.38 ± 0.17 5.43 ± 0.1 5.53 ± 0.18 5.06^a^ ± 0.23 5.45^a^ ± 0.12 5.04^a^ ± 0.23 5.48^a^ ± 0.1  WW 2011-12 6 ± 0.22 6.2 ± 0.19 6.25 ± 0.18 7.13 ± 0.31 **6.13^b^ ± 0.14 6.66^a^ ± 0.24 6.10^b^ ± 0.14 6.69^a^ ± 0.23**  Faba 2013 10.88 ± 0.35 13.75 ± 1.13 8.95 ± 0.94 14.3 ± 1.25 **9.91^b^ ± 0.59 14.03^a^ ± 0.79** 12.31^a^ ± 0.77 11.63^a^ ± 1.24  WW 2013-14 5.35 ± 0.29 7.23 ± 0.28 5.78 ± 0.36 6.65 ± 0.32 **5.56^b^ ± 0.23 6.94^a^ ± 0.23** 6.29^a^ ± 0.4 6.21^a^ ± 0.28  Maize 2015 6.6 ± 0.31 6.43 ± 0.53 6.3 ± 0.24 7 ± 0.21 6.45^a^ ± 0.19 6.71^a^ ± 0.29 6.51^a^ ± 0.29 6.65^a^ ± 0.2  P [g/kg] WW 2010-11 0.67 ± 0.05 0.62 ± 0.02 0.6 ± 0.02 0.61 ± 0.03 0.63^a^ ± 0.03 0.62^a^ ± 0.02 0.64^a^ ± 0.03 0.61^a^  ± 0.02  WW 2011-12 0.91 ± 0.06 1.03 ± 0.05 0.88 ± 0.02 1.08 ± 0.08 **0.89^b^ ± 0.03 1.06^a^ ± 0.04** 0.97^a^ ± 0.04 0.98^a^ ± 0.05  Faba 2013 1.5 ± 0.2 1.41 ± 0.27 1.12 ± 0.13 1.42 ± 0.18 1.31^a^ ± 0.13 1.42^a^ ± 0.15 1.45^a^ ± 0.16 1.27^a^ ± 0.12  WW 2013-14 0.89 ± 0.04 1.12 ± 0.07 0.79 ± 0.03 0.99 ± 0.06 **0.84^b^ ± 0.03 1.06^a^ ± 0.05 1.01^a^ ± 0.06 0.89^b^ ± 0.05**  Maize 2015 0.79 ± 0.03 0.83 ± 0.07 0.93 ± 0.05 0.95 ± 0.04 0.86^a^ ± 0.04 0.89^a^ ± 0.04 **0.81^b^ ± 0.04 0.94^a^ ± 0.03**  K [g/kg] WW 2010-11 2.57 ± 0.19 2.43 ± 0.43 2.84 ± 0.26 2.36 ± 0.28 2.70^a^ ± 0.16 2.40^a^ ± 0.24 2.50^a^ ± 0.22 2.60^a^ ± 0.2  WW 2011-12 7.73 ± 0.67 5.13 ± 0.19 8.5 ± 0.29 5.55 ± 0.23 **8.11^a^ ± 0.37 5.34^b^ ± 0.16** 6.43^a^ ± 0.59 7.02^a^ ± 0.58  Faba 2013 6.04 ± 0.78 4.72 ± 0.16 7.03 ± 0.77 4.05 ± 0.08 **6.54^a^ ± 0.54 4.39^b^ ± 0.15** 5.38^a^ ± 0.45 5.54^a^ ± 0.67  WW 2013-14 1.93 ± 0.01 1.99 ± 0.07 2.03 ± 0.06 1.95 ± 0.07 1.98^a^ ± 0.03 1.97^a^ ± 0.05 1.96^a^ ± 0.03 1.99^a^ ± 0.04  Maize 2015 10.18 ± 1.05 7.77 ± 1.06 10.24 ± 0.51 8.84 ± 0.6 10.21^a^ ± 0.54 8.30^a^ ± 0.6 8.97^a^ ± 0.83 9.54^a^ ± 0.45 |
