# Supplementary material for: Impact of crop residue management on crop production and soil chemistry after seven years of crop rotation in temperate climate, loamy soils
Source: PeerJ. 2018 May 23;6:e4836. doi: 10.7717/peerj.4836 (PMC5970559; doi:10.7717/peerj.4836)
Supplement: Table S6 — Significance code: ‘***’ p-value < 0.001; ‘**’ p-value < 0.01; ‘*’ p-value < 0.05. (Df: degree of freedom, Mean Sq: mean square). [file peerj-06-4836-s011.docx]

| **Nutrient Crop Df Mean Sq Fvalue Pvalue** |
| --- |
| **N** WW2010-11 Tillage 1 0.007656 4.143 0.088 .  Residue 1 0.006006 3.250 0.121  Tillage*Residue 1 0.002006 1.086 0.424  WW2011-12 Tillage 1 0.013806 14.826 0.00846 **  Residue 1 0.011556 12.409 0.01248 *  Tillage*Residue 1 0.004556 4.893 0.06896 .  Faba2013 Tillage 1 0.0189 0.310 0.5978  Residue 1 0.6765 11.091 0.0158 *  Tillage*Residue 1 0.0613 1.004 0.3550  WW2013-14 Tillage 1 0.00023 0.030 0.8674  Residue 1 0.07562 10.208 0.0187 *  Tillage*Residue 1 0.01000 1.350 0.2894  Maize2015 Tillage 1 0.000756 0.139 0.722  Residue 1 0.002756 0.508 0.503  Tillage*Residue 1 0.007656 1.412 0.280  **P** WW2010-11 Tillage 1 60.06 4.512 0.0778 .  Residue 1 12.60 0.947 0.3681  Tillage*Residue 1 26.52 1.993 0.2078  WW2011-12 Tillage 1 0.2 0.003 0.95496  Residue 1 1049.8 17.969 0.00545 **  Tillage*Residue 1 65.6 1.123 0.33004  Faba2013 Tillage 1 1340.3 0.789 0.409  Residue 1 447.1 0.263 0.626  Tillage*Residue 1 1621.2 0.954 0.366  WW2013-14 Tillage 1 510.8 15.897 0.007224 **  Residue 1 1840.4 57.283 0.000276 ***  Tillage*Residue 1 9.0 0.280 0.615619  Maize2015 Tillage 1 676.9 10.349 0.0182 *  Residue 1 39.0 0.597 0.4692  Tillage*Residue 1 0.8 0.013 0.9130  **K** WW2010-11 Tillage 1 383 0.242 0.6405  Residue 1 3688 2.325 0.1782  Tillage*Residue 1 1134 0.715 0.4302  WW2011-12 Tillage 1 14149 4.825 0.0704 .  Residue 1 307359 104.814 5.06e-05 ***  Tillage*Residue 1 1183 0.404 0.5487  Faba2013 Tillage 1 1028 0.046 0.8367  Residue 1 185001 8.341 0.0278 *  Tillage*Residue 1 27271 1.230 0.3100  WW2013-14 Tillage 1 38.44 0.345 0.579  Residue 1 3.80 0.034 0.860  Tillage*Residue 1 182.25 1.634 0.248  Maize2015 Tillage 1 12890 0.352 0.5744  Residue 1 146066 3.994 0.0926 .  Tillage*Residue 1 10162 0.278 0.6170 |
