# Supplementary material for: Impact of crop residue management on crop production and soil chemistry after seven years of crop rotation in temperate climate, loamy soils
Source: PeerJ. 2018 May 23;6:e4836. doi: 10.7717/peerj.4836 (PMC5970559; doi:10.7717/peerj.4836)
Supplement: Table S7 — Significance code: ‘***’ p-value < 0.001; ‘**’ p-value < 0.01; ‘*’ p-value < 0.05. (Df: degree of freedom, Mean Sq: mean square). [file peerj-06-4836-s012.docx]

| **Df Mean Sq Fvalue Pvalue** |
| --- |
| WW2009-10 Tillage 1 0.6 0.032 0.864  Residue 1 7.6 0.427 0.537  Tillage*Residue 1 55.0 3.109 0.128  WW2010-11 Tillage 1 0509.5 31.339 0.001 **  Residue 1 25.4 1.564 0.258  Tillage*Residue 1 0.1 0.005 0.946  WW2011-12 Tillage 1 5.1 0.124 0.737  Residue 1 33.1 0.810 0.403  Tillage*Residue 1 39.1 0.957 0.366  Faba2013 Tillage 1 1640.2 45.457 0.001 ***  Residue 1 49.0 1.358 0.288  Tillage*Residue 1 9.0 0.249 0.635  WW2013-14 Tillage 1 444.5 16.041 0.007 **  Residue 1 5.8 0.211 0.662  Tillage*Residue 1 242.8 8.763 0.025 *  Maize2015 Tillage 1 21.3 3.009 0.133  Residue 1 2.4 0.334 0.584  Tillage*Residue 1 5.3 0.752 0.419 |
| **Global rate Tillage 1 1403.7 29.95 4.83e-7** *** **Residue 1 100 2.134 0.148**  **Tillage*Residue 1 202.4 4.318 0.041** * |
