# Supplementary material for: Impact of crop residue management on crop production and soil chemistry after seven years of crop rotation in temperate climate, loamy soils
Source: PeerJ. 2018 May 23;6:e4836. doi: 10.7717/peerj.4836 (PMC5970559; doi:10.7717/peerj.4836)
Supplement: Table S8 — For each crop, treatments means with different letters are significantly different (ANOVA, p-value < 0.05). (WW: winter wheat, CT: conventional tillage, RT: reduced tillage, IN: incorporation of crop residue, OUT: exportation of crop residues). [file peerj-06-4836-s013.docx]

| Interaction between fixed factors No interaction between factors |
| --- |
| Crop residue management Residue fate Tillage type |
| CT-IN CT-OUT RT-IN RT-OUT IN OUT CT RT |
| WW 2010-05-20 3.65 ± 0.17 3.74 ± 0.19 3.26 ± 0.22 3.55 ± 0.12 **3.45^a^ ± 0.15 3.64^a^ ± 0.11 3.69^a^ ± 0.12 3.40^a^ ± 0.13**  2010-06-04 6.48 ± 0.39 6.90 ± 0.5 6.17 ± 0.03 7.04 ± 0.78 **6.32^a^ ± 0.19 6.97^a^ ± 0.43 6.69^a^ ± 0.31 6.60^a^ ± 0.4**  2010-06-25 7.53 ± 0.13 9.09 ± 0.33 7.78 ± 0.48 8.63 ± 0.21 **7.66^b^ ± 0.23 8.86^a^ ± 0.2 8.31^a^ ± 0.34 8.20^a^ ± 0.29**  2010-07-08 7.90 ± 0.26 8.40 ± 0.36 7.65 ± 0.54 7.82 ± 0.31 **7.77^a^ ± 0.28 8.11^a^ ± 0.24 8.15^a^ ± 0.23 7.73^a^ ± 0.29**  2010-07-22 5.92 ± 0.33 6.96 ± 0.32 5.60 ± 0.3 6.02 ± 0.3 **5.76^b^ ± 0.22 6.49^a^ ± 0.27 6.44^a^ ± 0.29 5.81^b^ ± 0.21**  2010-08-05 5.53 ± 0.26 6.39 ± 0.4 5.30 ± 0.21 6.30 ± 0.2 **5.41^b^ ± 0.16 6.35^a^ ± 0.21 5.96^a^ ± 0.27 5.80^a^ ± 0.23**  WW 2011-05-06 2.06 ± 0.22 2.38 ± 0.16 2.17 ± 0.17 2.28 ± 0.1 **2.11^a^ ± 0.13 2.33^a^ ± 0.09 2.22^a^ ± 0.14 2.22^a^ ± 0.09**  2011-05-20 4.72 ± 0.18 4.71 ± 0.3 4.30 ± 0.21 4.41 ± 0.35 **4.51^a^ ± 0.15 4.56^a^ ± 0.22 4.72^a^ ± 0.16 4.36^a^ ± 0.19**  2011-06-07 6.36 ± 0.18 6.89 ± 0.23 5.84 ± 0.21 6.47 ± 0.45 **6.10^b^ ± 0.16 6.68^a^ ± 0.25 6.62^a^ ± 0.17 6.15^b^ ± 0.26**  2011-06-23 6.39 ± 0.23 6.97 ± 0.54 5.28 ± 0.27 6.22 ± 0.12 **5.83^b^ ± 0.26 6.60^a^ ± 0.29 6.68^a^ ± 0.29 5.75^b^ ± 0.22**  2011-07-07 5.02 ± 0.18 5.8 ± 0.11 5.28 ± 0.38 5.32 ± 0.13 **5.15^b^ ± 0.2 5.56^a^ ± 0.12 5.41^a^ ± 0.18 5.30^a^ ± 0.18**  2011-07-20 4.99 ± 0.38 4.99 ± 0.2 4.52 ± 0.2 4.53 ± 0.07 **4.76^a^ ± 0.22 4.76^a^ ± 0.13 4.99^a^ ± 0.2 4.53^a^ ± 0.1**  2011-08-08 4.42 ± 0.24 4.28 ± 0.23 3.78 ± 0.21 3.95 ± 0.23 **4.10^a^ ± 0.19 4.11^a^ ± 0.16 4.35^a^ ± 0.15 3.86^b^ ± 0.15**  WW 2012-03-08 0.47 ± 0.03 0.48 ± 0.02 0.41 ± 0.04 0.43 ± 0.01 **0.44^a^ ± 0.03 0.46^a^ ± 0.02 0.48^a^ ± 0.02 0.42^a^ ± 0.02**  2012-04-19 1.10 ± 0.07 1.04 ± 0.1 1.01 ± 0.13 1.03 ± 0.02 **1.06^a^ ± 0.07 1.03^a^ ± 0.05 1.07^a^ ± 0.06 1.02^a^ ± 0.06**  2012-05-07 2.10 ± 0.16 2.27 ± 0.1 2.14 ± 0.27 1.82 ± 0.11 **2.12^a^ ± 0.14 2.05^a^ ± 0.11 2.18^a^ ± 0.09 1.98^a^ ± 0.15**  2012-05-23 5.88 ± 0.25 5.37 ± 0.48 4.65 ± 0.35 4.88 ± 0.27 **5.26^a^ ± 0.31 5.13^a^ ± 0.27 5.62^a^ ± 0.27 4.77^b^ ± 0.21**  2012-06-12 7.93 ± 0.41 9.13 ± 0.59 8.57 ± 0.65 8.38 ± 0.45 **8.25^a^ ± 0.38 8.76^a^ ± 0.37 8.53^a^ ± 0.4 8.48^a^ ± 0.37**  2012-06-27 9.83 ± 0.46 10.45 ± 0.49 11.07 ± 0.31 10.15 ± 0.57 **10.45^a^ ± 0.35 10.3^a^ ± 0.35 10.14^a^ ± 0.33 10.61^a^ ± 0.35**  2012-07-10 8.06 ± 0.43 9.28 ± 0.65 8.75 ± 0.34 8.64 ± 0.26 **8.41^a^ ± 0.28 8.96^a^ ± 0.34 8.67^a^ ± 0.43 8.69^a^ ± 0.2**  2012-08-07 6.90 ± 0.26 6.95 ± 0.35 6.47 ± 0.13 5.97 ± 0.25 **6.68^a^ ± 0.16 6.46^a^ ± 0.27 6.92^a^ ± 0.2 6.22^a^ ± 0.16**  Faba 2013-05-07 0.13 ± 0.01 0.11 ± 0 0.07 ± 0.01 0.07 ± 0 **0.10^a^ ± 0.01 0.09^a^ ± 0.01 0.12^a^ ± 0.01 0.07^b^ ± 0.01**  2013-05-27 **0.62^a^ ± 0.07 0.47^a^ ± 0.04 0.28^b^ ± 0.02 0.45^a^ ± 0.05** 0.45 ± 0.07 0.46 ± 0.03 0.55 ± 0.05 0.36 ± 0.04  2013-06-18 3.51 ± 0.22 3.6 ± 0.31 2.59 ± 0.35 2.29 ± 0.08 **3.05^a^ ± 0.26 2.94^a^ ± 0.29 3.56^a^ ± 0.18 2.44^b^ ± 0.17**  2013-07-04 7.06 ± 0.11 7.65 ± 1.21 6.02 ± 0.88 5.93 ± 0.39 **6.54^a^ ± 0.46 6.79^a^ ± 0.68 7.35^a^ ± 0.58 5.97^a^ ± 0.45**  2013-07-17 10.94 ± 0.51 12.0 ± 0.48 8.96 ± 1.11 10.58 ± 0.95 **9.95^a^ ± 0.68 11.29^a^ ± 0.56 11.47^a^ ± 0.38 9.77^a^ ± 0.74**  WW 2014-03-26 0.48 ± 0.01 0.37 ± 0.03 0.40 ± 0.06 0.44 ± 0.01 **0.44^a^ ± 0.03 0.41^a^ ± 0.02 0.43^a^ ± 0.02 0.42^a^ ± 0.03**  2014-04-10 1.05 ± 0.03 1.05 ± 0.09 1.02 ± 0.1 1.04 ± 0.08 **1.04^a^ ± 0.05 1.05^a^ ± 0.05 1.05^a^ ± 0.04 1.03^a^ ± 0.06**  2014-04-23 2.26 ± 0.02 2.38 ± 0.08 2.06 ± 0.14 2.18 ± 0.15 **2.16^a^ ± 0.07 2.28^a^ ± 0.09 2.32^a^ ± 0.04 2.12^a^ ± 0.1**  2014-05-06 4.37 ± 0.06 3.91 ± 0.5 3.77 ± 0.17 3.94 ± 0.29 **4.07^a^ ± 0.14 3.93^a^ ± 0.27 4.14^a^ ± 0.25 3.85^a^ ± 0.16**  2014-05-28 8.20 ± 0.61 8.37 ± 0.37 7.91 ± 0.09 7.83 ± 0.38 **8.05^a^ ± 0.29 8.10^a^ ± 0.27 8.28^a^ ± 0.33 7.87^a^ ± 0.18**  2014-06-20 10.32 ± 0.71 10.44 ± 0.24 9.59 ± 0.16 9.93 ± 0.19 **9.95^a^ ± 0.37 10.18^a^ ± 0.17 10.38^a^ ± 0.35 9.76^a^ ± 0.13**  2014-07-15 **8.03^a^ ± 0.43 8.24^a^ ± 0.31 8.04^a^ ± 0.46 6.56^b^ ± 0.21** 8.03 ± 0.29 7.40 ± 0.36 8.14 ± 0.25 7.30 ± 0.36  Maize 2015-07-06 3.77 ± 0.27 3.45 ± 0.29 2.55 ± 0.22 2.44 ± 0.29 **3.16^a^ ± 0.28 2.95^a^ ± 0.27 3.61^a^ ± 0.19 2.5^b^ ± 0.17**  2015-07-23 7.88 ± 0.34 7.39 ± 0.29 6.10 ± 0.4 5.67 ± 0.22 **6.99^a^ ± 0.41 6.53^a^ ± 0.37 7.63^a^ ± 0.23 5.88^b^ ± 0.23**  2015-09-17 8.14 ± 0.42 8.24 ± 0.25 6.71 ± 0.33 6.64 ± 0.26 **7.42^a^ ± 0.37 7.44^a^ ± 0.35 8.19^a^ ± 0.23 6.67^b^ ± 0.19**  2015-10-17 7.67 ± 0.42 7.20 ± 0.36 6.24 ± 0.33 6.39 ± 0.25 **6.96^a^ ± 0.37 6.80^a^ ± 0.25 7.44^a^ ± 0.27 6.31^b^ ± 0.19** |
