# Supplementary material for: Impact of crop residue management on crop production and soil chemistry after seven years of crop rotation in temperate climate, loamy soils
Source: PeerJ. 2018 May 23;6:e4836. doi: 10.7717/peerj.4836 (PMC5970559; doi:10.7717/peerj.4836)
Supplement: Table S9 — Significance code: ‘***’ p-value < 0.001; ‘**’ p-value < 0.01; ‘*’ p-value < 0.05; ‘.’ p-value < 0.1. (Df: degree of freedom, Mean Sq: mean square). [file peerj-06-4836-s014.docx]

| **Df Mean Sq F-value P-value** |
| --- |
| Winter wheat 2010-05-20 Tillage 1 0.3393 2.339 0.177  Residue 1 0.1425 0.982 0.360  Tillage*Residue 1 0.0390 0.269 0.623  2010-06-04 Tillage 1 0.0298 0.037 0.854  Residue 1 1.6706 2.081 0.199  Tillage*Residue 1 0.1958 0.244 0.639  2010-06-25 Tillage 1 0.046 0.325 0.5895  Residue 1 5.784 40.635 0.0007 ***  Tillage*Residue 1 0.497 3.492 0.1109  2010-07-08 Tillage 1 0.7014 1.040 0.347  Residue 1 0.4590 0.681 0.441  Tillage*Residue 1 0.1073 0.159 0.704  2010-07-22 Tillage 1 1.5750 11.419 0.01487 *  Residue 1 2.1170 15.349 0.00782 **  Tillage*Residue 1 0.3969 2.878 0.14074  2010-08-05 Tillage 1 0.101 0.612 0.46382  Residue 1 3.469 21.056 0.00374 **  Tillage*Residue 1 0.016 0.099 0.76406  Winter wheat 2011-05-06 Tillage 1 0.00003 0.000 0.984  Residue 1 0.19076 2.680 0.153  Tillage*Residue 1 0.03961 0.556 0.484  2011-05-20 Tillage 1 0.5172 1.697 0.240  Residue 1 0.0095 0.031 0.865  Tillage*Residue 1 0.0134 0.044 0.841  2011-06-07 Tillage 1 0.8832 9.467 0.02175 *  Residue 1 1.3385 14.348 0.00910 **  Tillage*Residue 1 0.0108 0.116 0.74498  2011-06-23 Tillage 1 3.422 17.819 0.00555 **  Residue 1 2.320 12.083 0.01321 *  Tillage*Residue 1 0.126 0.658 0.44816  2011-07-07 Tillage 1 0.0536 0.584 0.4738  Residue 1 0.6695 7.294 0.0355 *  Tillage*Residue 1 0.5450 5.938 0.0507 .  2011-07-20 Tillage 1 0.8649 4.216 0.0858 .  Residue 1 0.0003 0.001 0.9720  Tillage*Residue 1 0.0002 0.001 0.9738  2011-08-08 Tillage 1 0.9499 7.296 0.0355 *  Residue 1 0.0013 0.010 0.9245  Tillage*Residue 1 0.0899 0.691 0.4378  Winter wheat 2012-03-28 Tillage 1 0.011651 4.872 0.0694 .  Residue 1 0.001010 0.422 0.5398  Tillage*Residue 1 0.000007 0.003 0.9584  2012-04-19 Tillage 1 0.01001 0.271 0.621  Residue 1 0.00191 0.052 0.827  Tillage*Residue 1 0.00410 0.111 0.750  2012-05-07 Tillage 1 0.16144 2.418 0.1709  Residue 1 0.02136 0.320 0.5921  Tillage*Residue 1 0.24368 3.650 0.1046  2012-05-23 Tillage 1 2.9187 29.011 0.00169 **  Residue 1 0.0767 0.762 0.41620  Tillage*Residue 1 0.5427 5.394 0.05924 .  2012-06-12 Tillage 1 0.0118 0.009 0.929  Residue 1 1.0280 0.756 0.418  Tillage*Residue 1 1.9737 1.451 0.274  2012-06-27 Tillage 1 0.8816 1.197 0.316  Residue 1 0.0920 0.125 0.736  Tillage*Residue 1 2.4151 3.280 0.120  2012-07-10 Tillage 1 0.0018 0.003 0.961  Residue 1 1.2268 1.747 0.234  Tillage*Residue 1 1.7691 2.520 0.164  2012-08-07 Tillage 1 1.9966 8.796 0.0251 *  Residue 1 0.1998 0.880 0.3843  Tillage*Residue 1 0.3049 1.343 0.2905  Faba bean 2013-05-07 Tillage 1 0.011268 73.274 0.00014 ***  Residue 1 0.000230 1.493 0.26763  Tillage*Residue 1 0.000710 4.619 0.07520 .  2013-05-27 Tillage 1 0.13282 17.616 0.0057 **  Residue 1 0.00079 0.105 0.7568  Tillage*Residue 1 0.09781 12.973 0.0113 *  2013-06-18 Tillage 1 5.000 21.146 0.0037 **  Residue 1 0.046 0.194 0.6751 Tillage*Residue 1 0.147 0.623 0.4598  2013-07-04 Tillage 1 7.625 3.114 0.128  Residue 1 0.243 0.099 0.764  Tillage*Residue 1 0.473 0.193 0.676  2013-07-17 Tillage 1 11.544 2.459 0.168  Residue 1 7.177 1.528 0.263  Tillage*Residue 1 0.311 0.066 0.805  Winter wheat 2014-03-26 Tillage 1 0.000121 0.027 0.8752  Residue 1 0.004541 1.004 0.3551  Tillage*Residue 1 0.019612 4.335 0.0825 .  2014-04-10 Tillage 1 0.00124 0.092 0.7722  Residue 1 0.00028 0.021 0.8907  Tillage*Residue 1 0.00039 0.029 0.8708  2014-04-23 Tillage 1 0.16298 3.386 0.115  Residue 1 0.05567 1.157 0.323  Tillage*Residue 1 0.00000 0.000 0.997  2014-05-06 Tillage 1 0.3197 0.910 0.377  Residue 1 0.0794 0.226 0.651  Tillage*Residue 1 0.4070 1.158 0.323  2014-05-28 Tillage 1 0.6906 1.122 0.330  Residue 1 0.0092 0.015 0.907  Tillage*Residue 1 0.0596 0.097 0.766  2014-06-20 Tillage 1 1.5405 2.292 0.181  Residue 1 0.2165 0.322 0.591  Tillage*Residue 1 0.0455 0.068 0.803  2014-07-15 Tillage 1 2.7913 17.602 0.00571 **  Residue 1 1.6017 10.101 0.01912 *  Tillage*Residue 1 2.8253 17.816 0.00556 **  Maize 2015-07-06 Tillage 1 4.955 37.878 0.000844 ***  Residue 1 0.184 1.404 0.280925  Tillage*Residue 1 0.041 0.313 0.595951  2015-07-23 Tillage 1 12.222 27.121 0.002 **  Residue 1 0.857 1.902 0.217  Tillage*Residue 1 0.003 0.007 0.938  2015-09-17 Tillage 1 9.154 22.658 0.00313 **  Residue 1 0.001 0.002 0.96593  Tillage*Residue 1 0.030 0.074 0.79406  2015-10-14 Tillage 1 5.066 11.911 0.0136 *  Residue 1 0.103 0.242 0.6405 Tillage*Residue 1 0.381 0.895 0.3806 |
