# Supplementary material for: Impact of crop residue management on crop production and soil chemistry after seven years of crop rotation in temperate climate, loamy soils
Source: PeerJ. 2018 May 23;6:e4836. doi: 10.7717/peerj.4836 (PMC5970559; doi:10.7717/peerj.4836)
Supplement: Table S10 — For each crop, treatments means with different letters are significantly different (ANOVA, p-value < 0.05). (WW: winter wheat, CT: conventional tillage, RT: reduced tillage, IN: incorporation of crop residue, OUT: exportation of crop residues). [file peerj-06-4836-s015.docx]

| Interaction between fixed factors No interaction between factors |
| --- |
| Crop Date Crop residue management Residue fate Tillage type |
| CT-IN CT-OUT RT-IN RT-OUT IN OUT CT RT |
| WW 25/06/2010 2.29 ± 0.05 2.73 ± 0.07 2.43 ± 0.14 2.6 ± 0.05 **2.36^b^ ± 0.07 2.67^a^ ± 0.05 2.51^a^ ± 0.09 2.52^a^ ± 0.07**  8/07/2010 7.43 ± 0.36 7.5 ± 0.15 7.47 ± 0.38 6.82 ± 0.14 **7.45^a^ ± 0.24 7.16^a^ ± 0.16 7.47^a^ ± 0.18 7.14^a^ ± 0.22**  22/07/2010 10.23 ± 1.43 12.15 ± 0.38 10.88 ± 0.68 10.88 ± 0.63 **10.55^a^ ± 0.74 11.51^a^ ± 0.42 11.19^a^ ± 0.77 10.88^a^ ± 0.43**  5/08/2010 11.82 ± 0.49 12.82 ± 0.56 11.57 ± 0.36 12.68 ± 0.32 **11.69^b^ ± 0.28 12.75^a^  ± 0.3 12.32^a^ ± 0.39 12.12^a^ ± 0.31**  WW 7/06/2011 1.41 ± 0.04 1.53 ± 0.06 1.37 ± 0.07 1.51 ± 0.08 **1.39^b^ ± 0.04 1.52^a^ ± 0.05 1.47^a^ ± 0.04 1.44^a^ ± 0.05**  23/06/2011 2.98 ± 0.05 3.25 ± 0.28 2.59 ± 0.11 3.15 ± 0.04 **2.78^b^ ± 0.09 3.20^a^ ± 0.13 3.11^a^ ± 0.14 2.87^a^ ± 0.12**  7/07/2011 5.93 ± 0.11 6.73 ± 0.24 6.58 ± 0.37 6.69 ± 0.21 **6.25^a^ ± 0.22 6.71^a^ ± 0.15 6.33^a^ ± 0.19 6.64^a^ ± 0.2**  20/07/2011 8.79 ± 0.58 8.76 ± 0.38 8.39 ± 0.49 8.29 ± 0.17 **8.59^a^ ± 0.36 8.52^a^ ± 0.21 8.77^a^ ± 0.32 8.34^a^ ± 0.24**  8/08/2011 9 ± 0.5 8.76 ± 0.46 7.96 ± 0.41 8.67 ± 0.46 **8.48^a^ ± 0.36 8.71^a^ ± 0.3 8.88^a^ ± 0.32 8.31^a^ ± 0.32**  WW 12/06/2012 1.54 ± 0.11 1.71 ± 0.09 1.69 ± 0.11 1.63 ± 0.09 **1.61^a^ ± 0.08 1.67^a^ ± 0.06 1.62^a^ ± 0.07 1.66^a^ ± 0.07**  27/06/2012 3.2 ± 0.16 3.46 ± 0.2 3.89 ± 0.07 3.38 ± 0.2 **3.54^a^ ± 0.15 3.42^a^ ± 0.13 3.33^a^ ± 0.13 3.63^a^ ± 0.14**  10/07/2012 6.72 ± 0.57 7.2 ± 0.36 7.17 ± 0.34 6.86 ± 0.06 **6.95^a^ ± 0.32 7.03^a^ ± 0.18 6.96^a^ ± 0.33 7.02^a^ ± 0.17**  7/08/2012 10.88 ± 0.42 10.61 ± 0.52 10.39 ± 0.28 9.5 ± 0.34 **10.63^a^  ± 0.25 10.05^a^  ± 0.36 10.75^a^ ± 0.31 9.94^a^ ± 0.26**  Faba 4/07/2013 0.06 ± 0.02 0.1 ± 0.04 0.09 ± 0.03 0.16 ± 0.04 **0.08^a^ ± 0.02 0.13^a^ ± 0.03 0.08^a^ ± 0.02 0.13^a^ ± 0.03**  17/07/2013 2.44 ± 0.16 2.59 ± 0.33 1.97 ± 0.41 3.07 ± 0.44 **2.20^a^ ± 0.22 2.83^a^ ± 0.27 2.52^a^ ± 0.17 2.52^a^ ± 0.35**  WW 20/06/2014 3.15 ± 0.19 3.14 ± 0.09 3.3 ± 0.09 3.3 ± 0.06 **3.22^a^ ± 0.1 3.22^a^ ± 0.06 3.14^a^ ± 0.1 3.30^a^ ± 0.05**  15/07/2014 **9.8^a^ ± 0.38 9.71^a^ ± 0.52 10.29^a^ ± 0.47 8.43^b^ ± 0.18** 10.05 ± 0.3 9.07 ± 0.35 9.75 ± 0.3 9.36 ± 0.42  Maize 17/09/2015 4.26 ± 0.18 4.43 ± 0.1 3.83 ± 0.16 3.74 ± 0.06 **4.05^a^ ± 0.14 4.08^a^ ± 0.14 4.34^a^ ± 0.1 3.79^b^ ± 0.08**  14/10/2015 6.21 ± 0.46 6.92 ± 0.27 5.46 ± 0.25 5.93 ± 0.26 **5.83^a^ ± 0.28 6.43^a^ ± 0.25 6.57^a^ ± 0.28 5.69^b^ ± 0.19** |
