# Supplementary material for: Impact of crop residue management on crop production and soil chemistry after seven years of crop rotation in temperate climate, loamy soils
Source: PeerJ. 2018 May 23;6:e4836. doi: 10.7717/peerj.4836 (PMC5970559; doi:10.7717/peerj.4836)
Supplement: Table S11 — Significance code: ‘***’ p-value < 0.001; ‘**’ p-value < 0.01; ‘*’ p-value < 0.05; ‘.’ p-value < 0.1. (Df: degree of freedom, Mean Sq: mean square). [file peerj-06-4836-s016.docx]

| **Df Mean Sq F-value P-value** |
| --- |
| Winter wheat 2010-06-25 Tillage 1 0.39 0.064 0.809391  Residue 1 231.8 37.643 0.000858 ***  Tillage*Residue 1 61.45 9.979 0.019590 *  2010-07-08 Tillage 1 0.4128 1.321 0.294  Residue 1 0.3335 1.068 0.341  Tillage*Residue 1 0.5006 1.602 0.253  2010-07-22 Tillage 1 0.384 0.237 0.6435  Residue 1 3.667 2.263 0.1832  Tillage*Residue 1 3.686 2.274 0.1823  2010-08-05 Tillage 1 0.152 0.260 0.6281  Residue 1 4.452 7.623 0.0328 *  Tillage*Residue 1 0.013 0.023 0.8853  Winter wheat 2011-06-07 Tillage 1 0.00285 0.705 0.43321  Residue 1 0.06289 15.577 0.00757 **  Tillage*Residue 1 0.00065 0.160 0.70269  2011-06-23 Tillage 1 0.2391 2.551 0.1613  Residue 1 0.7036 7.508 0.0337 *  Tillage*Residue 1 0.0789 0.842 0.3942  2011-07-07 Tillage 1 0.3775 1.356 0.288  Residue 1 0.8269 2.971 0.136  Tillage*Residue 1 0.4744 1.705 0.239  2011-07-20 Tillage 1 0.7411 0.857 0.390  Residue 1 0.0191 0.022 0.887  Tillage*Residue 1 0.0048 0.006 0.943  2011-08-08 Tillage 1 1.2914 2.407 0.1717  Residue 1 0.2180 0.406 0.5473  Tillage*Residue 1 0.9125 1.701 0.2399  Winter wheat 2012-06-12 Tillage 1 0.00454 0.088 0.776  Residue 1 0.01134 0.220 0.655  Tillage*Residue 1 0.05476 1.065 0.342  2012-06-27 Tillage 1 0.3726 4.499 0.0782 .  Residue 1 0.0631 0.762 0.4163  Tillage*Residue 1 0.5862 7.079 0.0375 *  2012-07-10 Tillage 1 0.0128 0.017 0.900  Residue 1 0.0282 0.037 0.853  Tillage*Residue 1 0.6397 0.849 0.392  2012-08-07 Tillage 1 2.5829 4.255 0.0847 .  Residue 1 1.3439 2.214 0.1873  Tillage*Residue 1 0.3993 0.658 0.4483  Faba bean 2013-07-04 Tillage 1 0.008345 2.066 0.201  Residue 1 0.011892 2.944 0.137  Tillage*Residue 1 0.001257 0.311 0.597  2013-07-17 Tillage 1 0.008345 2.066 0.201  Residue 1 0.011892 2.944 0.137  Tillage*Residue 1 0.001257 0.311 0.597  Winter wheat 2014-06-20 Tillage 1 0.09890 1.574 0.256  Residue 1 0.00011 0.002 0.968  Tillage*Residue 1 0.00011 0.002 0.968  2014-07-15 Tillage 1 0.612 1.710 0.2388  Residue 1 3.801 10.620 0.0173 *  Tillage*Residue 1 3.162 8.833 0.0249 *  Maize 2015-09-17 Tillage 1 1.2332 17.439 0.00584 **  Residue 1 0.0057 0.081 0.78534  Tillage*Residue 1 0.0696 0.985 0.35937  2015-10-14 Tillage 1 3.0624 7.551 0.0334 *  Residue 1 1.4114 3.480 0.1114  Tillage*Residue 1 0.0549 0.135 0.7255 |
