# Supplementary material for: Impact of crop residue management on crop production and soil chemistry after seven years of crop rotation in temperate climate, loamy soils
Source: PeerJ. 2018 May 23;6:e4836. doi: 10.7717/peerj.4836 (PMC5970559; doi:10.7717/peerj.4836)
Supplement: Table S12 — Significance code: ‘***’ p-value < 0.001; ‘**’ p-value < 0.01; ‘*’ p-value < 0.05; ‘.’ p-value < 0.1. (Df: degree of freedom, Mean Sq: mean square). [file peerj-06-4836-s017.docx]

| **Df Mean Sq F-value P-value** |
| --- |
| Winter wheat 2010-05-20 Tillage 1 0.3393 2.339 0.177  Residue 1 0.1425 0.982 0.360  Tillage*Residue 1 0.0390 0.269 0.623  2010-06-04 Tillage 1 0.0298 0.037 0.854  Residue 1 1.6706 2.081 0.199  Tillage*Residue 1 0.1958 0.244 0.639  2010-06-25 Tillage 1 0.041 0.181 0.68500  Residue 1 9.136 40.421 0.00071 ***  Tillage*Residue 1 0.965 4.271 0.08427 .  2010-07-08 Tillage 1 2.1978 1.350 0.289  Residue 1 0.0105 0.006 0.939  Tillage*Residue 1 1.0764 0.661 0.447  2010-07-22 Tillage 1 3.516 1.394 0.2823  Residue 1 11.357 4.505 0.0780 .  Tillage*Residue 1 6.503 2.579 0.1594  2010-08-05 Tillage 1 0.494 0.375 0.5626  Residue 1 15.860 12.062 0.0133 *  Tillage*Residue 1 0.061 0.047 0.8363  Winter wheat 2011-05-06 Tillage 1 0.00003 0.000 0.984  Residue 1 0.19076 2.680 0.153  Tillage*Residue 1 0.03961 0.556 0.484  2011-05-20 Tillage 1 0.5172 1.697 0.240  Residue 1 0.0095 0.031 0.865  Tillage*Residue 1 0.0134 0.044 0.841  2011-06-07 Tillage 1 0.03967 8.919 0.02443 *  Residue 1 0.07082 15.923 0.00720 **  Tillage*Residue 1 0.00076 0.171 0.69354  2011-06-23 Tillage 1 5.470 10.086 0.0192 *  Residue 1 5.580 10.287 0.0184 *  Tillage*Residue 1 0.405 0.747 0.4207  2011-07-07 Tillage 1 0.1466 0.240 0.6413  Residue 1 2.9844 4.894 0.0689 .  Tillage*Residue 1 2.0364 3.339 0.1174  2011-07-20 Tillage 1 3.207 1.740 0.235  Residue 1 0.015 0.008 0.931  Tillage*Residue 1 0.003 0.002 0.970  2011-08-08 Tillage 1 4.456 3.799 0.0992 .  Residue 1 0.253 0.215 0.6590  Tillage*Residue 1 1.575 1.343 0.2906  Winter wheat 2012-03-28 Tillage 1 0.011651 4.872 0.0694 .  Residue 1 0.001010 0.422 0.5398  Tillage*Residue 1 0.000007 0.003 0.9584  2012-04-19 Tillage 1 0.01001 0.271 0.621  Residue 1 0.00191 0.052 0.827  Tillage*Residue 1 0.00410 0.111 0.750  2012-05-07 Tillage 1 0.16144 2.418 0.1709  Residue 1 0.02136 0.320 0.5921  Tillage*Residue 1 0.24368 3.650 0.1046  2012-05-23 Tillage 1 2.9187 29.011 0.00169 **  Residue 1 0.0767 0.762 0.41620  Tillage*Residue 1 0.5427 5.394 0.05924 .  2012-06-12 Tillage 1 0.0017 0.001 0.977  Residue 1 1.2552 0.650 0.451  Tillage*Residue 1 2.6860 1.392 0.283  2012-06-27 Tillage 1 2.400 1.936 0.2135  Residue 1 0.307 0.248 0.6362  Tillage*Residue 1 5.381 4.341 0.0823 .  2012-07-10 Tillage 1 0.024 0.009 0.927  Residue 1 1.627 0.621 0.461 Tillage*Residue 1 4.536 1.731 0.236  2012-08-07 Tillage 1 9.121 5.880 0.0515 .  Residue 1 2.580 1.663 0.2446  Tillage*Residue 1 1.402 0.904 0.3785  Faba bean 2013-05-07 Tillage 1 0.011268 73.274 0.00014 ***  Residue 1 0.000230 1.493 0.26763  Tillage*Residue 1 0.000710 4.619 0.07520 .  2013-05-27 Tillage 1 0.13282 17.616 0.0057 **  Residue 1 0.00079 0.105 0.7568  Tillage*Residue 1 0.09781 12.973 0.0113 *  2013-06-18 Tillage 1 5.000 21.146 0.0037 **  Residue 1 0.046 0.194 0.6751 Tillage*Residue 1 0.147 0.623 0.4598  2013-07-04 Tillage 1 7.129 2.763 0.148  Residue 1 0.362 0.140 0.721  Tillage*Residue 1 0.425 0.165 0.699  2013-07-17 Tillage 1 11.562 1.299 0.298  Residue 1 15.462 1.737 0.236  Tillage*Residue 1 2.271 0.255 0.631  Winter wheat 2014-03-26 Tillage 1 0.000121 0.027 0.8752  Residue 1 0.004541 1.004 0.3551  Tillage*Residue 1 0.019612 4.335 0.0825 .  2014-04-10 Tillage 1 0.00124 0.092 0.7722  Residue 1 0.00028 0.021 0.8907  Tillage*Residue 1 0.00039 0.029 0.8708  2014-04-23 Tillage 1 0.16298 3.386 0.115  Residue 1 0.05567 1.157 0.323  Tillage*Residue 1 0.00000 0.000 0.997  2014-05-06 Tillage 1 0.3197 0.910 0.377  Residue 1 0.0794 0.226 0.651  Tillage*Residue 1 0.4070 1.158 0.323  2014-05-28 Tillage 1 0.6906 1.122 0.330  Residue 1 0.0092 0.015 0.907  Tillage*Residue 1 0.0596 0.097 0.766  2014-06-20 Tillage 1 0.8587 0.770 0.414  Residue 1 0.2067 0.185 0.682  Tillage*Residue 1 0.0501 0.045 0.839  2014-07-15 Tillage 1 6.018 6.718 0.0411 *  Residue 1 10.337 11.541 0.0145 *  Tillage*Residue 1 11.964 13.357 0.0106 *  Maize 2015-07-06 Tillage 1 4.955 37.878 0.000844 ***  Residue 1 0.184 1.404 0.280925  Tillage*Residue 1 0.041 0.313 0.595951  2015-07-23 Tillage 1 12.222 27.121 0.002 **  Residue 1 0.857 1.902 0.217  Tillage*Residue 1 0.003 0.007 0.938  2015-09-17 Tillage 1 17.108 22.036 0.00335 **  Residue 1 0.011 0.014 0.90985  Tillage*Residue 1 0.191 0.246 0.63728  2015-10-14 Tillage 1 16.007 9.859 0.0201 *  Residue 1 0.752 0.463 0.5214 Tillage*Residue 1 0.146 0.090 0.7740 |
