# Supplementary material for: Impact of crop residue management on crop production and soil chemistry after seven years of crop rotation in temperate climate, loamy soils
Source: PeerJ. 2018 May 23;6:e4836. doi: 10.7717/peerj.4836 (PMC5970559; doi:10.7717/peerj.4836)
Supplement: Table S13 — Significance code: ‘***’ p-value < 0.001; ‘**’ p-value < 0.01; ‘*’ p-value < 0.05. (Df: degree of freedom, Mean Sq: mean square). [file peerj-06-4836-s018.docx]

| **Df Mean Sq F-value P-value** |
| --- |
| Winter wheat Tillage 1 0.7560 2.636 0.156  2009-2010 Residue 1 0.0525 0.183 0.684  Tillage*Residue 1 0.1475 0.514 0.500  Winter wheat Tillage 1 1.5968 44.093 0.000563 ***  2010-11 Residue 1 2.1445 59.215 0.000252 *** Tillage*Residue 1 0.0340 0.939 0.369991  Winter wheat Tillage 1 0.22691 2.032 0.204  2011-12 Residue 1 0.00476 0.043 0.843  Tillage*Residue 1 0.01761 0.158 0.705  Faba bean 2013 Tillage 1 0.05454 0.979 0.3606  Residue 1 0.02407 0.432 0.5353 Tillage*Residue 1 0.02914 0.523 0.4967  Winter wheat Tillage 1 0.04796 0.794 0.407  2013-14 Residue 1 0.00003 0.001 0.982  Tillage*Residue 1 0.01717 0.284 0.613  Maize 2015 Tillage 1 0.8651 8.863 0.0247 *  Residue 1 0.1671 1.712 0.2386 Tillage*Residue 1 0.1744 1.787 0.2298 |
| **TOTAL Tillage 1 9.238 13.540 0.0103 ***  **Residue 1 1.451 2.126 0.1951**  **Tillage*Residue 1 0.000 0.000 0.9831** |
