# Supplementary material for: Impact of crop residue management on crop production and soil chemistry after seven years of crop rotation in temperate climate, loamy soils
Source: PeerJ. 2018 May 23;6:e4836. doi: 10.7717/peerj.4836 (PMC5970559; doi:10.7717/peerj.4836)
Supplement: Table S14 — For each crop, treatments means with different letters are significantly different (ANOVA, p < 0.05). (WW: winter wheat, CT: conventional tillage, RT: reduced tillage, IN: incorporation of crop residue, OUT: exportation of crop residues). [file peerj-06-4836-s019.docx]

| Interaction between fixed factors No interaction between factors |
| --- |
| Nutrient Crop Crop residue management Residue fate Tillage type |
| CT-IN CT-OUT RT-IN RT-OUT IN OUT CT RT |
| N [g/kg] WW 2010-11 17.18 ± 0.29 16.8 ± 0.25 17.75 ± 0.26 17.1 ± 0.15 17.46^a^ ± 0.21 16.95^a^ ± 0.15 16.99^a^ ± 0.19 17.43^a^ ± 0.19  WW 2011-12 16.4 ± 0.5 17.28 ± 0.06 17.33 ± 0.23 16.38 ± 0.63 16.86^a^ ± 0.31 16.83^a^ ± 0.34 16.84^a^ ± 0.29 16.85^a^ ± 0.36  Faba 2013 40.08 ± 0.61 39.58 ± 0.41 39.15 ± 1.15 40.7 ± 0.16 39.61^a^ ± 0.63 40.14^a^ ± 0.3 39.83^a^ ± 0.35 39.93^a^ ± 0.61  WW 2013-14 20.88 ± 0.15 20.9 ± 0.26 20.88 ± 0.27 20.4 ± 0.31 20.88^a^ ± 0.14 20.65^a^ ± 0.21 20.89^a^ ± 0.14 20.64^a^ ± 0.21  Maize 2015 12.98 ± 0.21 12.48 ± 0.13 12.3 ± 0.48 12.3 ± 0.36 12.64^a^ ± 0.27 12.39^a^ ± 0.18 12.73^a^ ± 0.15 12.3^a^ ± 0.28  P [g/kg] WW 2010-11 2.78 ± 0.02 2.7 ± 0.03 2.8 ± 0.03 2.69 ± 0.06 **2.79^a^ ± 0.02 2.7^b^ ± 0.03** 2.74^a^ ± 0.02 2.75^a^ ± 0.04  WW 2011-12 2.93 ± 0.04 2.91 ± 0.05 2.87 ± 0.05 2.93 ± 0.02 2.9^a^ ± 0.03 2.92^a^ ± 0.03 2.92^a^ ± 0.03 2.90^a^ ± 0.03  Faba 2013 5.58 ± 0.07 5.72 ± 0.07 5.57 ± 0.14 5.58 ± 0.15 5.58^a^ ± 0.07 5.65^a^ ± 0.08 5.65^a^ ± 0.05 5.57^a^ ± 0.09  WW 2013-14 3.55 ± 0.14 3.47 ± 0.05 3.51 ± 0.08 3.38 ± 0.13 3.53^a^ ± 0.07 3.42^a^ ± 0.07 3.51^a^ ± 0.07 3.44^a^ ± 0.07  Maize 2015 2.25 ± 0.27 2.16 ± 0.09 2.03 ± 0.14 2.26 ± 0.13 2.14^a^ ± 0.15 2.21^a^ ± 0.08 2.20^a^ ± 0.13 2.15^a^ ± 0.1  K [g/kg] WW 2010-11 3.78 ± 0.03 3.74 ± 0.04 3.87 ± 0.04 3.84 ± 0.1 3.82^a^ ± 0.03 3.79^a^ ± 0.05 3.76^a^ ± 0.02 3.86^a^ ± 0.05  WW 2011-12 3.82 ± 0.2 4.03 ± 0.24 4.25 ± 0.08 4.22 ± 0.09 4.03^a^ ± 0.13 4.13^a^ ± 0.12 **3.92^b^ ± 0.15 4.23^a^ ± 0.06**  Faba 2013 11.42 ± 0.23 11.35 ± 0.31 11.5 ± 0.3 11.17 ± 0.2 11.46^a^ ± 0.18 11.26^a^ ± 0.18 11.39^a^ ± 0.18 11.33^a^ ± 0.18  WW 2013-14 3.66 ± 0.19 3.66 ± 0.08 3.82 ± 0.15 3.58 ± 0.1 3.74^a^ ± 0.11 3.62^a^ ± 0.06 3.66^a^ ± 0.09 3.70^a^ ± 0.09  Maize 2015 3.47 ± 0.2 3.39 ± 0.12 3.65 ± 0.18 3.79 ± 0.26 3.56^a^ ± 0.13 3.59^a^ ± 0.15 **3.43^b^ ± 0.11 3.72^a^ ± 0.15** |
